# Lighting the way: an economical alternative to feeder cell irradiation for T-cell expansion

**DOI:** 10.3389/fimmu.2024.1453740

**Published:** 2024-09-11

**Authors:** Michael Benavidez Arias, An Nguyen, Daniel Ross, David Eagerton, Krit Ritthipichai

**Affiliations:** Department of Biomedical Affairs and Research, Edward Via College of Osteopathic Medicine, Spartanburg, SC, United States

**Keywords:** UVC irradiation, feeder cells, T-cell expansion, T-cell therapy, TILs

## Abstract

A robust T-cell expansion process involves co-culturing T-cells with non-proliferating feeder cells combined with anti-CD3 antibody and IL-2. Although ionizing irradiation effectively inhibits feeder cell proliferation, the high operating costs limit cell therapy research to well-funded institutions. UVC, known for causing DNA damage-induced cell death and commonly used for environmental sterilization, presents a cost-effective alternative to ionizing irradiation for generating non-proliferating feeder cells. UVC irradiation of K562 artificial antigen presenting cells (aAPCs) resulted in significant DNA damage, evidenced by increased γ-H2AX phosphorylation within 15 minutes and elevated 8-OHdG levels at 24 hours. This indicates the occurrence of DNA double-strand breaks and oxidative damage. Following UVC irradiation, glucose uptake and ATP production were significantly reduced, whereas aCD3 retention at the surface of the cell increased twofold. Selective inhibition of glucose uptake and ATP production similarly enhanced aCD3 retention by approximately 10-fold and 6-fold, respectively. This suggests that UVC-induced energy deprivation dampens aCD3 internalization, potentially enhancing T-cell activation through prolonged aCD3 and T-cell receptor interaction. Tumor-infiltrating lymphocytes (TILs) expanded with UVC-irradiated PBMCs demonstrated comparable viability, expansion, immunophenotype, and effector function to those expanded with ionizing irradiation. UVC irradiation was equally effective in suppressing feeder cell proliferation and facilitating the expansion of functionally potent T-cells compared to traditional ionizing irradiation. Implementing UVC irradiation in T-cell expansion can significantly reduce costs, enhancing the accessibility and feasibility of cell therapy research across various institutions.

## Introduction

Cancer cell therapy has emerged as a promising treatment for both hematologic and solid cancers. With seven FDA-approved cell therapeutic products currently in use for B-cell malignancies, the efficacy of these treatments is underscored by an impressive average objective response rate (ORR) ranging from 40 to 98% (1). Furthermore, over two thousand clinical trials involving cell therapy are underway, reflecting significant academic and industrial expansion in this field over the past decade (2). The success of cell manufacturing hinges on the complex process of immune cell expansion, which includes cell isolation, genetic modifications, and cell activation/proliferation (3). This process requires advanced laboratory facilities, specialized equipment, and a highly skilled team, posing substantial challenges for smaller research institutes with limited infrastructure and resources.

Efficient T-cell expansion necessitates robust T-cell activation (signal 1), optimal co-stimulation (signal 2), and adequate growth factors (signal 3). Strategies for T-cell expansion can be categorized into cell-dependent and cell-independent methods. Cell-independent approaches utilize human T-activator beads, soluble T-cell activators, or plate-bound T-cell activators (4). In contrast, cell-dependent approaches employ feeder cells such as peripheral blood mononuclear cells (PBMCs) or artificial antigen-presenting cells (aAPCs) (5, 6). Although both strategies stimulate T-cell expansion via anti-CD3 activation, cell-based approaches more closely mimic physiological T-cell activation by engaging various natural co-stimulatory ligands expressed by feeder cells, typically resulting in higher fold expansion (7). However, the proliferation of feeder cells can compete for essential nutrients like glucose, amino acids, and vitamins, potentially affecting T-cell expansion (8). To ensure efficient T-cell expansion, it is crucial to suppress feeder cell proliferation. This can be achieved through chemotherapeutic drug treatment or ionization irradiation. Chemotherapeutic drugs impede cell proliferation by targeting DNA replication during the G1, S, and G2 phases of the cell cycle (9). Despite being an inexpensive and convenient method, residual drug presence in the culture media can hinder immune cell expansion. In contrast, ionizing radiation, such as gamma rays and X-rays, induces irreparable DNA double-strand breaks, leading to cell apoptosis (10). Although both methods stop cell division, ionizing radiation is generally more effective at generating non-proliferating feeder cells and does not interfere with T-cell proliferation. Due to the high operational costs of ionizing irradiators, cell therapy research is predominantly conducted in well-equipped research institutes or relies on expensive commercially available irradiated feeder cells (11). To broaden the scope of cell therapy research, it is crucial to develop alternative methods for generating non-proliferating feeder cells, particularly for laboratories with limited access to irradiated cells.

Ultraviolet (UV) ray, a type of electromagnetic wave, offers a potential alternative. The UV spectrum, characterized by wavelengths shorter than visible light (400-700 nm) and longer than X-rays (<100 nm), includes UVA (315-400 nm), UVB (280-315 nm), and UVC (200-280 nm) (12). Among these, UVC, with its shortest wavelength and highest energy, causes the most severe DNA damage and exhibits potent germicidal properties. UVC irradiation has demonstrated the ability to impede cell proliferation and induce apoptosis (13, 14). Given that UVC generators are 100 times more cost-effective than ionizing irradiators, we investigated their potential for generating non-proliferating feeder cells for T-cell expansion (11). Despite the existing use of UV radiation in feeder cell generation, critical aspects such as the specific UV type, optimal irradiation method, and impact on feeder cells remain unaddressed (15, 16).

Our results showed that UVC-induced DNA damage disrupted cellular energy production, consequently impeding endocytosis. This disruption led to increased retention of antibodies on the surface of feeder cells, potentially sustaining early-phase T-cell activation. Notably, T-cells expanded with UVC-irradiated feeder cells demonstrated comparable fold expansion, immunophenotypic profiles, and effector function to those expanded with ionization-irradiated feeder cells. Therefore, UVC irradiation may serve as a convenient and cost-effective alternative for generating feeder cells for T-cell expansion. By circumventing the limitations of ionizing irradiators, this method could alleviate barriers faced by smaller research institutions, fostering advancements in T-cell therapy research and development.

## Materials and methods

### Generation of CD32hi K562 cells

To generate the PB CD32 vector, the human CD32 sequence was inserted into the PB-CMV-MCS-EF1α-GreenPuro plasmid (System Biosciences). K562 cells (1 × 10^6^ cells) were electroporated with 1 µg of the PB CD32 vector and 0.4 µg of the Super piggyBac Transposase expression vector (System Biosciences) using the Neon™ Electroporation System (Invitrogen). Post-electroporation, the cells were selected by culturing in media containing puromycin (10 µg/ml) for 21 days. The selected CD32hi K562 cells were then assessed for their antibody binding affinity. The cells were stained with anti-human CD3 antibody (clone OKT3, Biolegend) at varying concentrations, ranging from 0.1 to 1000 ng, and analyzed by Novocyte 1000 (Agilent).

### UVC irradiation

CD32hi K562 or wild-type (WT) K562 cells (1 × 10^6^ cells/ml) were seeded into each well of a four-well plate. The cells were subjected to UVC irradiation at a dose of 10,000 µJ/cm^2^ using a Fisherbrand™ UV Crosslinker (ThermoFisher Scientific) and immediately incubated in a cell incubator. The cells were utilized for downstream experiments including cell viability assessment, apoptosis assay, γ-H2AX phosphorylation analysis, 8-OHdG DNA quantification, ATP measurement, and glucose uptake.

### K562 cell viability and apoptosis assay

K562 cells (1 × 10^6^ cells/ml) were irradiated with UVC at a dose of 10,000 µJ/cm^2^. The cells were stained with trypan blue and counted daily for 14 days using the Invitrogen™ Countess™ 3 Automated Cell Counter System (ThermoFisher Scientific). On day 14, apoptosis was assessed using the Annexin V Apoptosis Detection Kit with 7-AAD (BioLegend). Immediately after staining, the cells were analyzed using the Novocyte 1000.

### Detection of γ-H2AX phosphorylation

The irradiated K562 cells (1 × 10^6^ cells) were intranuclear stained with Mouse Anti-H2AX (pS139) antibody (clone N1-431, BD Biosciences) using the eBioscience™ Intracellular Fixation & Permeabilization Buffer Set (ThermoFisher Scientific). The cells were immediately acquired using the Novocyte 1000.

### 8-OHdG DNA damage quantification

Genomic DNA was extracted from K562 cells (3 × 10^6 cells) 24 hours after radiation using the DNeasy Blood & Tissue Kits (Qiagen). The extracted genomic DNA (300 ng) was assessed for oxidative damage using the EpiQuik™ 8-OHdG DNA Damage Quantification Direct Kit (Epigentek). The colorimetric signal was detected at 450 nm using the BioTek Gen5 Microplate Reader (BioTek Instruments).

### ATP assay

CD32hi K562 cells were irradiated with UVC at a dose of 10,000 µJ/cm^2^ using a Fisherbrand™ UV Crosslinker. Post-irradiation, the cells were harvested at 6 and 24 hours. Intracellular ATP levels were then evaluated using the ATP Assay Kit (Dojindo). The luminescent signal generated from the ATP assay was measured using the BioTek Gen5 Microplate Reader.

### Glucose uptake

CD32hi K562 cells were harvested at 6 and 24 hours post-UVC irradiation to evaluate glucose uptake. The glucose uptake was assessed using the Glucose Uptake-Glo™ Assay (Promega). The luminescent signal was measured using the BioTek Gen5 Microplate Reader.

### Antibody retention assay

An antibody retention assay was conducted on CD32hi K562 cells irradiated with UVC, treated with cytochalasin B (0 to 30 nM), rotenone (0 to 30 nM), or cultured in glucose-free media. The cells were initially incubated with anti-human CD3 antibody (clone OKT3, BioLegend) at 4°C for 20 minutes and then washed with FACS buffer. To initiate internalization, the cells were incubated at 37°C in a cell incubator for 25 minutes. Following this, the cells were washed again with FACS buffer and stained with anti-mouse IgG antibody (clone Poly4060, BioLegend) at 4°C for 20 minutes. After a final wash with FACS buffer, the cells were acquired using the Novocyte 1000. The percentage of antibody retention was determined by comparing the percentage of positive cells before and after the internalization process.

### Tumor-infiltrating lymphocyte expansion

Lung tumor specimens were obtained from lung cancer patients through the Cooperative Human Tissue Network (CHTN) under an Institutional Review Board-approved protocol (IRB#2021-027). Tumor fragments were dissected into 1 to 2 mm^3^ pieces. Each fragment was cultured in TIL culture media (RPMI (Gibco^®^) supplemented with 10% heat-inactivated Human AB serum (Valley Biomedical), 10 mM HEPES (Gibco^®^), 0.05 mM 2-Mercaptoethanol (Gibco^®^), 2 mM GlutaMAX™ Supplement (Gibco^®^), 1 mM sodium pyruvate (Gibco^®^)) and cGMP rHu IL-2 (6000 IU/ml) (Akronbio) for 14 days. Subsequently, TILs were further propagated using a Rapid Expansion Protocol (REP). TILs (2x10^4^ cells) were co-cultured with UVC-irradiated human PBMCs (2x10^6^ cells) (iQ Biosciences) or X-ray-irradiated human PBMCs (2x10^6^ cells) (iQ Biosciences), in combination with anti-human CD3 antibody (30 ng/ml) and IL-2 (3000 IU/ml). The cells were expanded in TIL culture media for 11 days before being utilized for various analyses, including cell count and viability assessment, immunophenotypic characterization, and effector function assays.

### Irradiation of PBMCs

To generate UVC-irradiated cells, cryopreserved human PBMCs (iQ Biosciences) were thawed in TIL culture media and rested for two hours. Ten million cells were plated into 60 mm-plate at 2e6 cells per ml and irradiated with UVC at a dose of 30 µJ/cm^2^ using a Fisherbrand™ UV Crosslinker. The irradiated cells were then used for TIL propagation using REP. The commercially cryopreserved irradiated human PBMCs (iQ Biosciences), which were treated with X-ray Irradiation at 25 Gy, were thawed in TIL culture media and rested for two hours prior to use for TIL expansion.

### T-cell effector function analysis

Post-REP TILs were re-stimulated with plate-bound anti-human CD3 OKT3 (100 ng/ml) and anti-human CD28 (50 ng/ml). After 24 hours, the supernatants were collected to quantify the level of IFN-γ using the IFN gamma Human ELISA Kit (ThermoFisher Scientific), with absorbance measured at 450 nm using the BioTek Gen5 Microplate Reader. In a separate experiment, cells were re-stimulated with plate-bound anti-human CD3 OKT3 (100 ng/ml) and anti-human CD28 (50 ng/ml) for 6 hours and treated with Brefeldin A (BioLegend) and anti-human CD107a (clone H4A3, BioLegend). Subsequently, the cells were analyzed using the Novocyte 1000 within 3 hours after the addition of Brefeldin A and anti-human CD107a.

### Immunophenotype and TCR Vβ analysis

For comprehensive immunophenotypic characterization, TILs were stained for various surface markers including CD3(clone UCHT1, Tonbo Biosciences), CD4(clone RPA-4, Tonbo Biosciences), CD8 (clone RPA-8, Tonbo Biosciences), CD28 (clone 28.2, Tonbo Biosciences), CD45RA (clone HI100, BioLegend), CD56(clone HCD56, Biolegend), CCR7(clone G043H7, BioLegend), KLRG1(clone 2F1/KLRG1), PD-1 (clone EH12.2H7, BioLegend), LAG-3 (clone 7H2C65, BioLegend), and TIM-3(clone A18087E, BioLegend). Additionally, to assess the TCR Vβ repertoire, cells were stained for CD3 (clone UCHT1, Tonbo Biosciences) and 24 antibody clones representing different TCR Vβ families using the Beta Mark TCR Vβ repertoire kit (Beckman Coulter). All stained samples were analyzed using the Novocyte 1000.

### Glucose consumption

The extracellular glucose in the supernatants was measured using the GlucCell^®^ Glucose Monitoring System (Esco VacciXcell). The percentage of glucose consumption was calculated by comparing the initial glucose concentration (mg/dL) in the culture media with the remaining glucose concentration after treatments.

### Statistical analysis

Graphs, plots, and data analyses were performed using GraphPad Prism 9 Software (GraphPad). Data are presented as mean ± standard error of the mean (SEM). Statistical significance was determined using two-tailed Student’s t-tests.

## Results

### UVC radiation induces DNA double-strand breaks and apoptosis in K562 aAPCs

**Figure 1:**
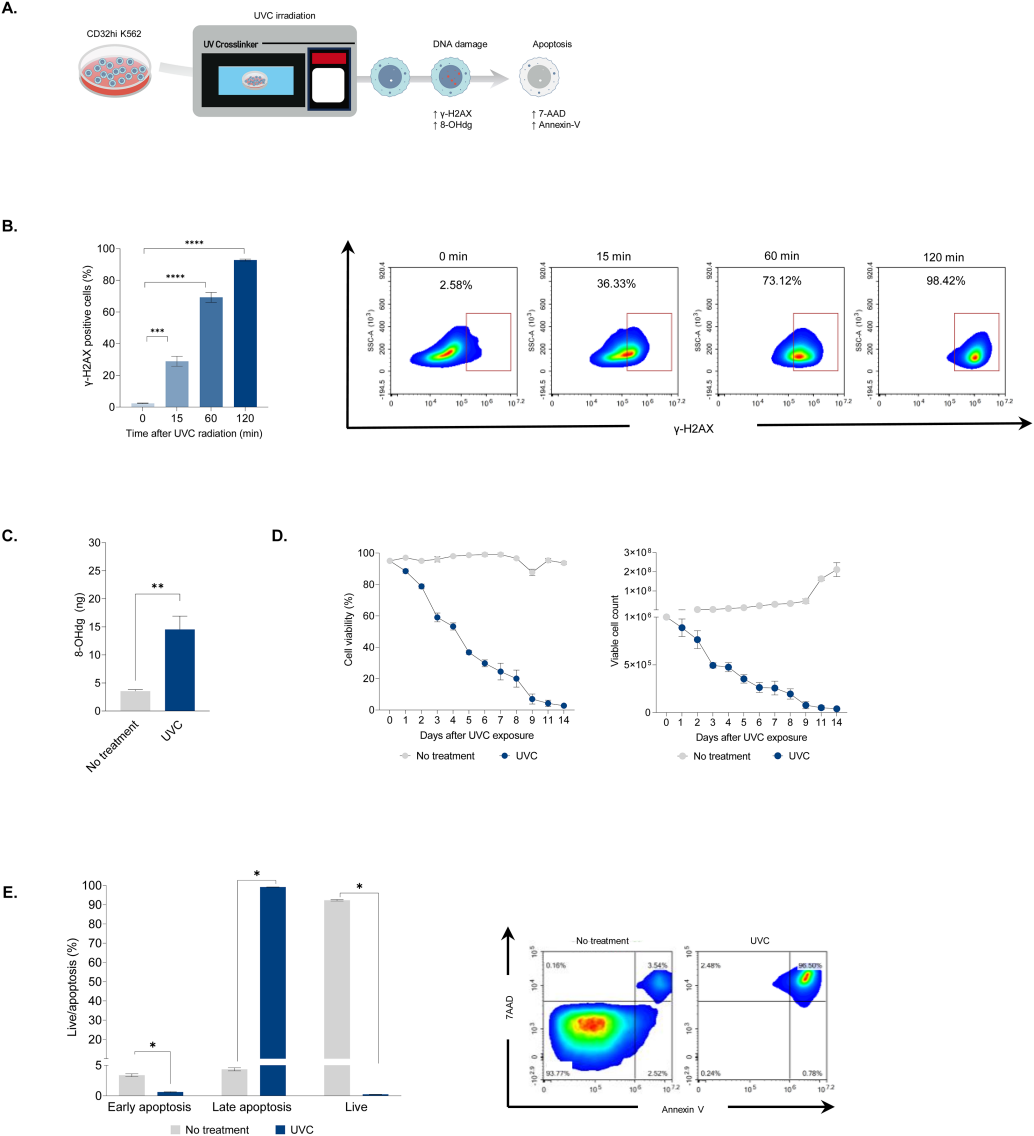
The impact of UVC irradiation on DNA damage and apoptosis. **(A)** Schematic illustrating the assessment of CD32hi K562 cells following UVC exposure. The cells were irradiated with UVC at a dose of 10,000 μJ/cm2 and evaluated for DNA damage, cell viability, and cell death. **(B)** Flow cytometry analysis of phosphorylated γ-H2AX in CD32hi K562 cells 15, 60, and 120 minutes after UVC irradiation (left) and representative density plots corresponding to the kinetics of phosphorylated γ-H2AX (right). **(C)** ELISA for the level of 8-Ohdg in UVC irradiated CD32hi K562 cells 24 hours following UVC exposure. **(D)** Multiple line graphs demonstrating cell viability (left) and cell count (right) in CD32hi K562 pre- and post-UVC irradiation from day 0 to 14. **(E)** Bar graph showing cell apoptosis in CD32hi K562 cells on day 14 post-UVC irradiation (left) and representative flow cytometry plots of cell apoptosis, defined by 7AAD (late apoptosis) and Annexin V (early apoptosis) (right). Data are presented as mean ± SEM. Two independent experiments; n=5, per group. Two-tailed Student's t-test; *P ≤ 0.05; **P ≤ 0.01; ***p ≤ 0.001 ****p ≤ 0.0001.

Ionizing irradiation has been shown to induce DNA double-strand breaks (DSBs) in a dose-dependent manner, leading to irreversible DNA damage and subsequent apoptosis (10). On the other hand, UVC irradiation primarily causes pyrimidine dimerization, disrupting DNA replication and resulting in mutations after DNA repair (17). To further elucidate the effects of UVC irradiation on DNA damage and cellular function, we evaluated DNA damage, cell viability, and apoptosis in K562 artificial antigen presenting cells (aAPCs) cells following UVC irradiation (**Figure 1A**). To investigate the kinetics of DNA DSBs in K562 aAPCs, we examined γ-H2AX phosphorylation by flow cytometry. DNA damage was evident in approximately 30% of cells within 15 minutes of UVC irradiation. By 120 minutes, over 90% of the cells exhibited phosphorylated γ-H2AX (**Figure 1B**). This result demonstrates significant DNA damage comparable to that caused by ionizing irradiation (18). Both ionizing and UVC irradiation generate oxidative stress, leading to the formation of reactive oxygen species (ROS) that damage lipids, cell membranes, proteins, and nucleotides (19). After 24 hours of UVC exposure, a three-fold increase in 8-OHdG levels was observed, indicating oxidative DNA damage (**Figure 1C**). This indicates that despite the differences in wavelength and energy, UVC irradiation can induce oxidative damage similar to that caused by UVA, UVB, and ionizing irradiation (20). The co-stimulatory signals provided by feeder cells are crucial for early T-cell activation. However, proliferating feeder cells can interfere with T-cell proliferation by outcompeting nutrients and oxygen. Ionizing irradiation has been shown to effectively halt cell proliferation in feeder cells (8). To assess the impact of UVC irradiation on cell proliferation and apoptosis, K562 cell viability and expansion were monitored for 14 days post-irradiation. Viability declined from 90% to 50% within the first five days and dropped below 1% by day 14 (**Figure 1D**, left). Correspondingly, non-irradiated cells proliferated nearly 200-fold, while UVC-irradiated cell numbers decreased from 1x 10^6^ to below 2 x 10^4^ (**Figure 1D**, right). Flow cytometry analysis revealed that approximately 98% of UVC-irradiated cells underwent apoptosis, predominantly late apoptosis (7AAD^+^Annexin V^+^), while untreated cells maintained high viability at more than 95% (7AAD^-^Annexin V^-^) (**Figure 1E**). These results demonstrate that UVC irradiation effectively induces cell death similarly to ionizing irradiation.

### UVC irradiation enhances antibody retention by dampening cellular energy metabolism

**Figure 2:**
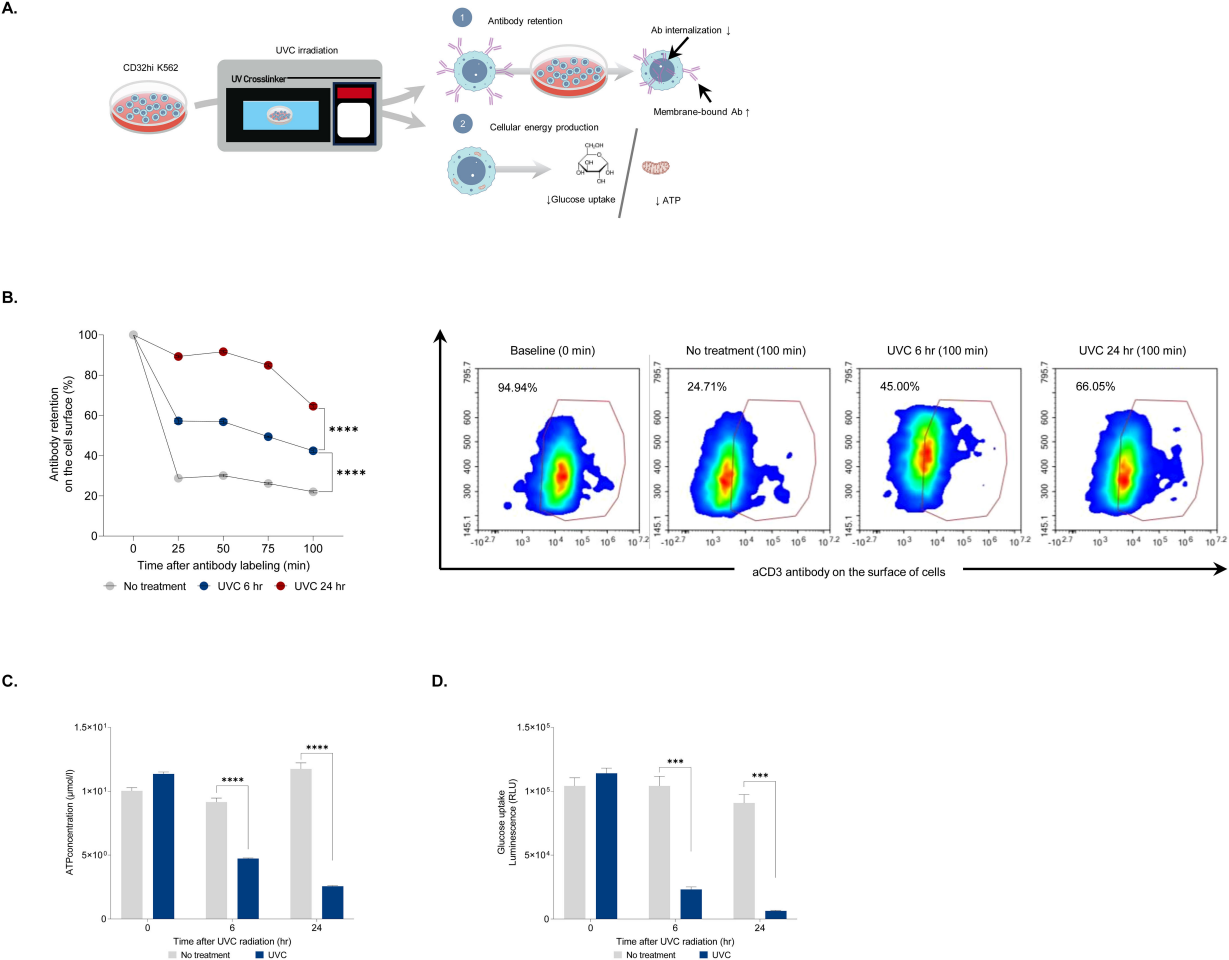
Assessment of antibody retention after UVC irradiation. **(A)** An experimental design for evaluating antibody retention in CD32hi K562 cells after UVC irradiation. The cells were irradiated with UVC and assessed for membrane-bound antibody retention, glucose uptake, and ATP level. **(B)** Bar graph displaying the percentage of membrane-bound antibodies on cells exposed to UVC for 6 and 24 hours at 25, 50, 75, and 100 minutes after antibody labeling (left) and representative flow cytometry plots demonstrating the level of the antibody in UVC-irradiated CD32hi K562 cells at 0 and 100 minutes after labeling with aCD3 antibody (right). **(C)** Bar graph demonstrating ATP concentrations at 6 and 24 hours after UVC irradiation. **(D)** Luminescence assay for measuring glucose uptake at 6 and 24 hours post-UVC treatment. Data are presented as mean ± SEM. Two independent experiments; n=5, per group. Two-tailed Student's t-test; ***p ≤ 0.001 ****p ≤ 0.0001.

A potent T-cell rapid expansion protocol (REP) involves co-culturing T-cells with non-proliferating feeder cells, anti-human CD3 antibody (aHuCD3), and IL-2. PBMCs-derived feeder cells, which consist of various immune cells such as monocytes and lymphocytes, provide essential co-stimulatory signals and cytokines for optimal T-cell expansion (6). Additionally, Fc receptors on feeder cells bind to aHuCD3, directly triggering T-cell activation (21). We hypothesized that UVC irradiation impacts energy generation, thereby impeding Fc receptor-mediated endocytosis. To test this, we generated K562 aAPCs that constitutively express CD32, a FcγRII receptor, using the PiggyBac transposon system. K562 aAPCs with high CD32 expression (CD32hi K562) showed a binding affinity for aHuCD3 antibodies approximately seven times greater than unmodified K562 aAPCs (**Supplementary Figure 1**). The cells were used to assess antibody retention and cellular energy production post-UVC irradiation (**Figure 2A**). UVC-irradiated CD32hi K562 cells were labeled with aHuCD3 antibodies and incubated at 37°C for 100 minutes. Antibody retention in untreated cells decreased from 100% to 30% within 25 minutes, whereas UVC-irradiated cells exhibited higher retention (60% and 90% at 6- and 24-hour post-irradiation, respectively) (**Figure 2B**). After 50 minutes, antibody retention in UVC-irradiated cells remained at least one-fold higher than in untreated cells, suggesting impaired endocytosis (**Figure 2B**). Adenosine triphosphate (ATP) is a key energy source synthesized by both glycolysis and mitochondrial respiration (22). Receptor-mediated endocytosis, involving the uptake of extracellular molecules into cells, is a major ATP-dependent cellular process (23). We hypothesized that UVC irradiation might impair energy production, leading to weakened endocytosis. To explore this link, ATP levels were measured in CD32hi K562 cells following UVC irradiation. A 2.5- and 4.5-fold decrease in intracellular ATP was observed at 6 and 24 hours post-UVC irradiation, respectively (**Figure 2C**). Since glucose is one of the most common sources of ATP production, glucose uptake was measured at 6 and 24 hours after UVC irradiation. The cells were incubated with 2-deoxyglucose (2DG), and NADPH was measured by luciferase activity. Compared to non-UVC-irradiated cells, an approximately 15-fold decrease in luminescence activity was evident 24 hours post-UVC irradiation (**Figure 2D**). In addition, a significant correlation between the percentage of glucose uptake and the level of ATP strengthened our hypothesis that decreased energy production may interrupt antibody internalization (**Supplementary Figure 2**).

### Inhibition of glucose uptake and ATP synthesis interrupts receptor-mediated endocytosis

**Figure 3:**
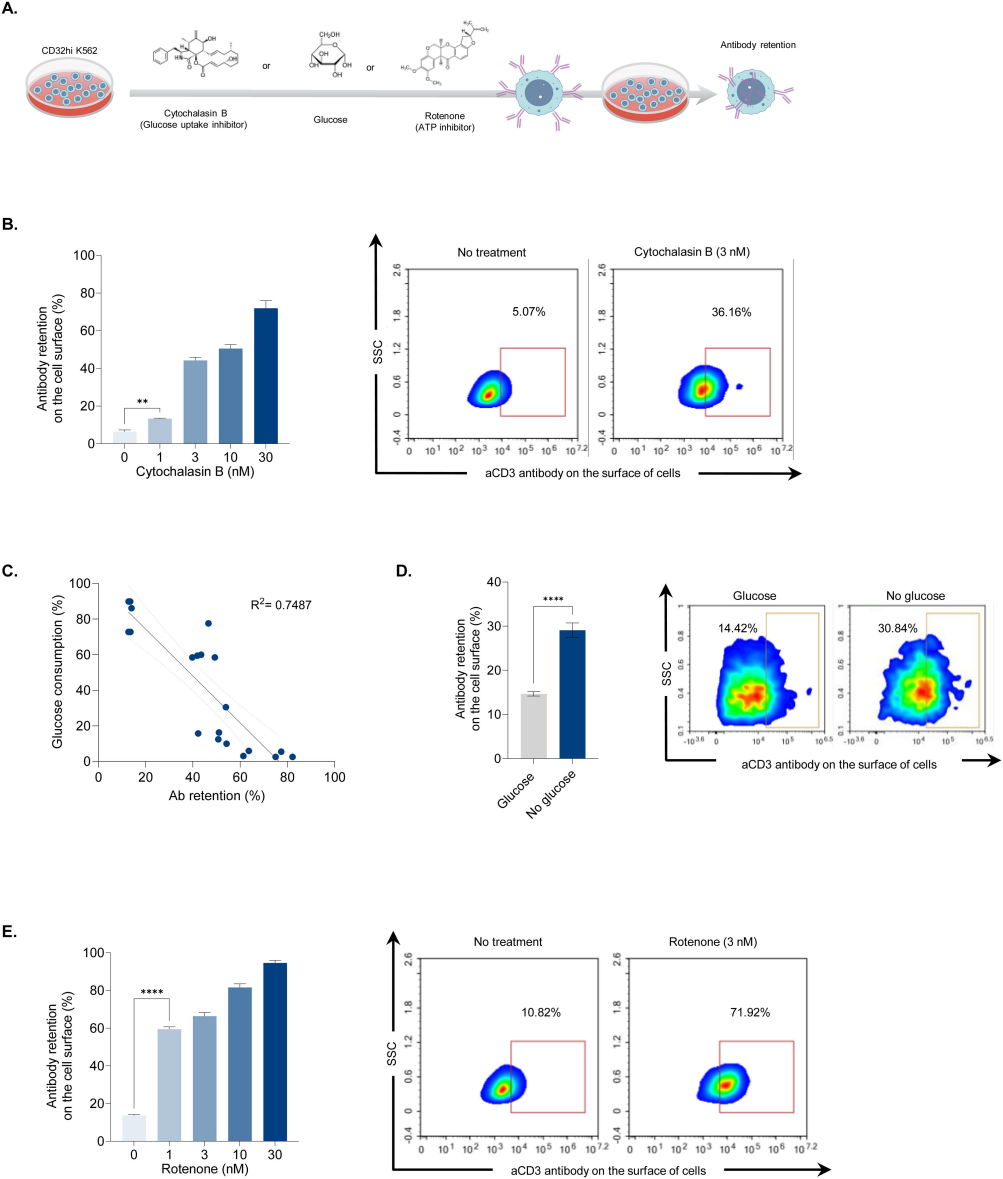
Association between antibody retention and cellular energy depletion. **(A)** Illustration showing antibody retention and glucose consumption analyses following treatments that disrupted cellular energy production. CD32hi K562 cells were treated with cytochalasin B (0 to 30 nM), rotenone (0 to 30 nM), or cultured in glucose-free media. After 24 hours, the cells were examined for membrane-bound antibodies and extracellular glucose levels. **(B)** Bar graph demonstrating the effect of cytochalasin B treatment on antibody retention post-antibody labeling (left) and representative density plots illustrating the percentage of membrane-bound antibodies in cells treated with cytochalasin B at 3 nM (right). **(C)** Scatter plot displaying a correlation coefficient between glucose consumption and antibody retention. **(D)** Bar graph showing antibody retention when glucose is depleted (left) and representative flow cytometry plots displaying the percentage of the remaining antibody on the cell surface after labeling. **(E)** Bar graph depicting antibody retention in rotenone-treated cells (left) and representative density plots showing the percentage of surface antibodies following rotenone treatment at 3 nM (right). Data are shown as mean ± SEM. Two independent experiments; n=5, per group. Two-tailed Student's t-test; **P ≤ 0.01 ****P ≤ 0.0001. R^2^ between 0.7 and 0.9 considered highly correlated.

While we observed a link between increased antibody retention and decreased cellular energy following UVC irradiation, this correlation does not establish causation. Therefore, we conducted additional experiments to investigate the causal relationship between cellular energy production and antibody retention. We used two chemical inhibitors that selectively suppress glucose uptake (cytochalasin B) and ATP production (rotenone) (**Figure 3A**). CD32hi cells were incubated with cytochalasin B at concentrations ranging from 0 to 30 nM and assessed for antibody retention. The effect of cytochalasin B was observed at 1 nM and increased in a dose-dependent manner. At the highest concentration (30 nM), antibody retention reached 75%, a four-fold increase compared to the untreated control (**Figure 3B**). Additionally, cells treated with cytochalasin B showed decreased extracellular glucose uptakes as the concentration increased. A strong negative correlation (r^2^ = 0.74) between glucose consumption and antibody retention was noted following cytochalasin treatment, indicating that glucose may be essential for antibody internalization (**Figure 3C**). To confirm whether glucose deprivation leads to increased antibody retention, CD32hi cells cultured in glucose-free media were tested for antibody retention. These cells exhibited twice the antibody retention on the cell surface compared to those supplemented with glucose, suggesting that glucose is one of the energy sources for Fc receptor-mediated endocytosis (**Figure 3D**). Next, we examined whether blocking ATP production would interfere with antibody internalization. Using rotenone at concentrations between 1 and 30 nM, cells were assessed for antibody retention. A significant increase in antibody retention on the cell surface was observed at a concentration of 1 nM, with almost 60% of cells displaying antibodies on the surface. At the lowest concentration (1 nM), the antibody retention induced by rotenone (ATP production inhibitor) was approximately four times higher than that caused by cytochalasin B (glucose uptake inhibitor), reinforcing the notion that endocytosis is an ATP-dependent process (**Figure 3E**). These findings support our hypothesis that UVC irradiation impairs glucose uptake and ATP production, consequently hindering antibody internalization.

### UVC irradiated cells can be used for TIL expansion

**Figure 4:**
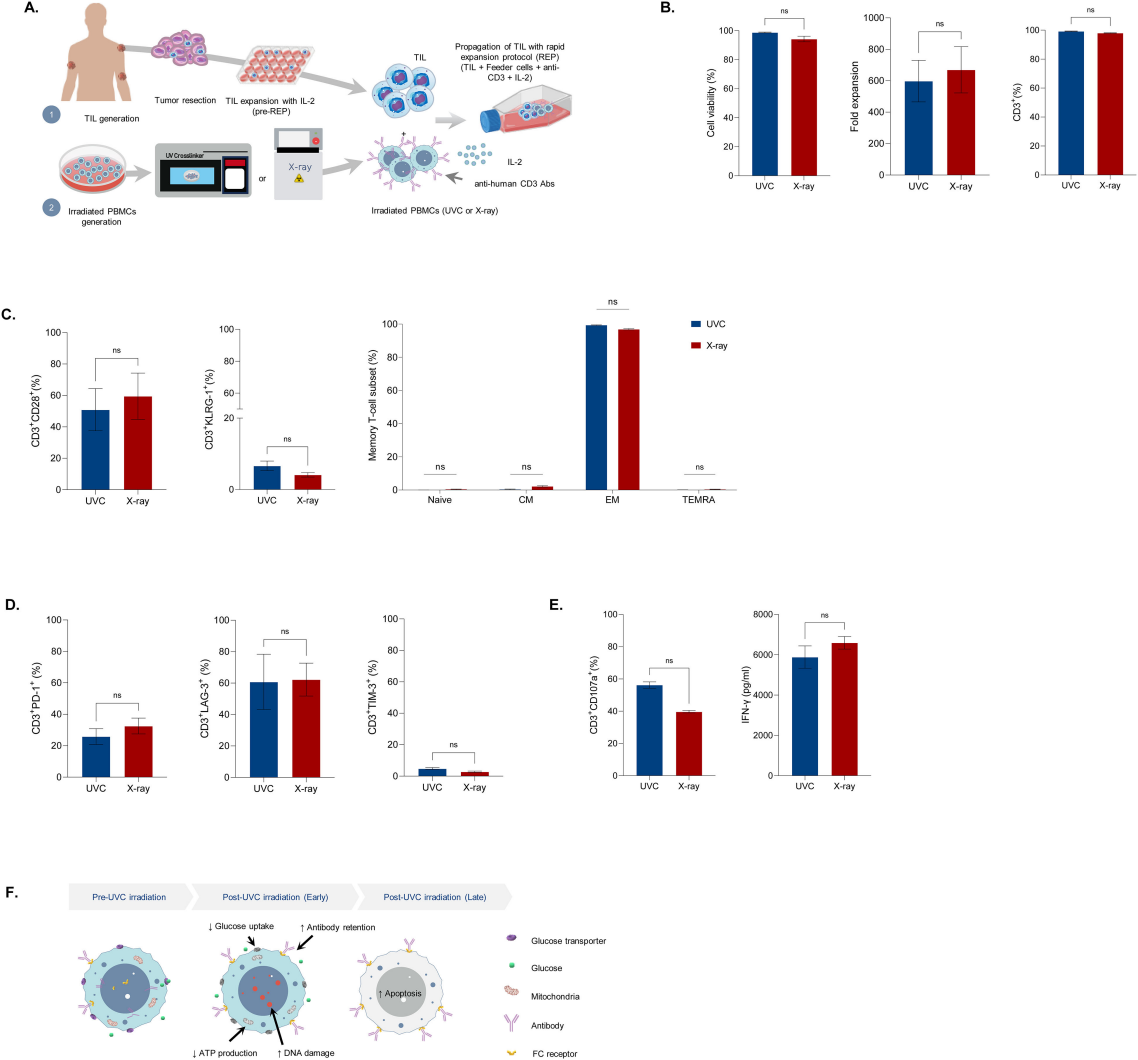
Characteristics of Tumor-infiltrating lymphocytes (TILs) expanded with irradiated feeder cells. **(A)** Diagram of TIL generation. TILs were grown from resected lung tumors with IL-2 (6000 IU/ml) for 14 days. The cells were further propagated with a rapid expansion protocol (REP) through co-culture of One TILs with anti-CD3 (30 ng/ml), IL-2 (3000 IU/ml), and UVC-irradiated PBMCs or X-ray irradiated PBMCs. On day 11, the cells were assessed for cell count, viability, immunophenotype, and T-cell effector function upon re-stimulation with anti-CD3/CD28. **(B)** Bar graphs depicting live cells (left), cell expansion (middle), and T-cell purity (right). **(C)** Bar graphs illustrating T-cell differentiation status (left and middle) and memory T-cell subsets of expanded Two TILs (right). **(D)** Bar graphs displaying T-cell exhaustion markers in expanded TILs. **(E)** Bar graphs showing TIL effector function following re-stimulation with anti-human CD3/CD28 antibodies. **(F)** Schematic illustrating altered metabolism and function of cells after UVC exposure. UVC irradiation resulted in increased DNA and decreased glucose uptake and ATP production, leading to enhanced antibody retention and cell apoptosis. Data are presented as mean ± SEM. Two independent experiments; n=4, per group. Two-tailed Student's t-test, ns; not significant.

As UVC irradiation stimulated cell apoptosis and enhanced antibody retention in feeder cells, it shows potential for use in T-cell expansion. To investigate the potential of UVC-irradiated PBMCs in enhancing T-cell expansion, we initially expanded tumor-infiltrating lymphocytes (TILs) for 14 days using IL-2, followed by an additional 11 days of rapid expansion (REP) with IL-2 and aHuCD3 in combination with UVC-irradiated or X-ray irradiated PBMCs. The cells were then harvested for analysis of cell count, viability, immunophenotype, and effector function (**Figure 4A**). TILs from both processes exhibited high cell viability (>90%) and similar levels of cell expansion, approximately 600-fold (**Figure 4B**, left and middle). The majority of TILs derived from both methods were CD3^+^ T-cells (>95%), with only a small fraction of CD3^-^CD56^+^ NK cells (0.1%) and CD3^-^CD56^-^ cells (<5%) (**Figure 4B** right, **Supplementary Figure 3**). Immunophenotypic characterization revealed that TILs expanded from both processes had comparable differentiation status, with similar percentages of CD28 and KLRG1 positive T-cells (**Figure 4C** left, **Supplementary Figure 3**). Most memory T-cells (>97%) were effector cells (CD45^-^CCR7^-^) (**Figure 4C** right, **Supplementary Figure 3**). Additionally, TILs expanded with UVC-irradiated PBMCs did not show increased exhaustion, as indicated by comparable levels of PD-1, LAG-3, and TIM-3 (**Figure 4D**, **Supplementary Figure 3**). To assess effector function, we measured CD107 expression and IFN-γ secretion in cells restimulated with aHuCD3. TILs expanded by both processes demonstrated approximately 50% degranulation and high IFN-γ production (~6000 pg/ml) (**Figure 4E**). Additionally, the level of CD107a in unstimulated cells and the fold increase in CD107a following anti-CD3/CD28 stimulation were comparable (**Supplementary Figure 4**). These results indicate that the key quality attributes, including purity, identity, potency, and immunophenotype, were comparable between the two processes. Overall, our study demonstrated the biological impact of UVC on cell damage and apoptosis. UVC irradiation disrupts ATP production and glucose uptake due to DNA damage, resulting in diminished cell endocytosis. The retention of antibodies on the surface of the cells prolongs T-cell activation during the early stages, leading to enhanced proliferation of T-cells (**Figure 4F**). These findings suggest that UVC irradiation could serve as a cost-effective and efficient alternative to ionizing irradiation for TIL expansion, maintaining the quality attributes necessary for effective T-cell function.

## Discussion

While previous studies have explored the use of UV radiation in feeder cell generation, our findings offer a deeper understanding of how UVC affects DNA damage and energy production, leading to antibody retention on the cell surface prior to apoptosis. Our results demonstrate that UVC irradiation not only effectively attenuates feeder cell proliferation but also promotes T-cell expansion comparable to ionizing irradiation. This equivalency extends across key parameters, including cell viability, T-cell expansion, immunophenotype, and T-cell effector function, highlighting the potential of UVC irradiation as a robust alternative in T-cell expansion method.

A previous study has demonstrated that ionizing irradiation causes rapid DNA damage, leading to the phosphorylation of γ-H2AX within 60 minutes (24). Our findings showed that UVC irradiation induces similar DNA damage but at a slower rate, taking nearly 120 minutes to achieve comparable levels. Although DNA damage by UVC irradiation appeared to delay relative to ionizing irradiation, we observed that UVC irradiation mediated apoptosis was effective in both CD32hi K562 cells and human PBMCs, with more than 97% of cells undergoing apoptosis by day 11 post-irradiation (**Figure 1E**, **Supplementary Figure 5**). This suggests that UVC, having lower energy than ionizing radiation, results in less immediate but sufficiently potent DNA damage and apoptosis over time. Thus, UVC is an effective method for generating non-proliferating feeder cells.

DNA damage from X-ray irradiation triggers cell cycle arrest and reduces energy production by disrupting ATP synthesis and glucose uptake (25). Our study revealed that UVC irradiation significantly hampers cellular energy production, leading to diminished antibody internalization by Fc receptors. While glucose is a source of ATP production, cells treated with cytochalasin B, a glucose uptake inhibitor, exhibited approximately four times less antibody retention compared to those treated with rotenone, an ATP production inhibitor. This observation suggests that blocking ATP production rather than glucose uptake is more effective in preventing endocytosis, thereby increasing antibody retention. Thus, we conclude that glucose is not the main source of ATP production in these cells. Generally, ATP production from lipids is the most efficient, yielding over 100 ATP molecules per fatty acid. In contrast, ATP production from glucose and amino acids results in approximately 36 and 30 ATP molecules, respectively. Recent studies have demonstrated that certain cancer cells, such as Pancreatic Ductal Adenocarcinoma and K562 cells, preferentially utilize fatty acid oxidation over glycolysis for ATP production (26)​. Thus, this impaired internalization is likely due to ATP deprivation, as supported by our findings that a significant decrease in intracellular ATP led to increased antibody retention on UVC-irradiated feeder cells. This enhanced retention supports sustained T-cell activation, crucial for the robust expansion of clonal T-cells during the initial stages of the rapid expansion process (REP) (7, 27).

During REP, TCR repertoire distribution may be altered, with some TCR repertoires expanding more than others. Differentiating tumor-reactive TILs from non-tumor-reactive ones remains challenging due to the polyclonal nature of TILs, MHC diversity, and the limitation of immune repertoire profiling technologies. Nevertheless, our results demonstrated that UVC-irradiated PBMCs did not alter clonal distribution or skew the ratio of CD4/CD8 T-cell subsets. The comparable TCR Vβ family distribution and CD4/CD8 ratio in TILs expanded with UVC and X-ray-irradiated PBMCs indicate that UVC irradiation preserves the clonality of TILs (**Supplementary Figure 6**). While both UVC and ionizing irradiation produced TILs with high IFN-γ production and efficient degranulation, this study did not assess tumor reactivity or perform killing assays due to the absence of autologous tumor cells. Further research is necessary to confirm whether TILs expanded with UVC-irradiated PBMCs exhibit comparable tumor recognition and tumor cell lysis functions to those expanded with X-ray-irradiated PBMCs.

Given the limited number of TIL lines used, it is essential to validate this process across various tumor indications to ensure its broader applicability. TILs undergo significant expansion during the traditional REP, achieving approximately a 1000-fold increase. However, because the REP in this study was conducted on a small scale, it yielded a lower fold expansion (~600-fold) compared to conventional REP processes (5). Thus, further process development studies must be conducted to scale up from small-research scale TIL expansion to larger scales.

Despite the growth in T-cell therapy over the past decade, most studies rely on expensive ionizing irradiators available only at well-funded research institutions. Our method using a cost-effective UVC crosslinker enables T-cell expansion in laboratories lacking ionizing irradiators, potentially making cell therapy research more accessible to all. This economical T-cell expansion approach can catalyze advancements in identifying novel TCRs, discovering neoantigens, and revolutionizing NK/T-cell expansion, paving the way for a new era in cell therapy research and application. By providing a feasible and cost-effective alternative to ionizing irradiation, UVC irradiation can expand the scope and accessibility of T-cell therapy research, driving innovation and therapeutic development across diverse research settings.

## Data Availability

The raw data supporting the conclusions of this article will be made available by the authors, without undue reservation.
