# Supplementary material for: Lighting the way: an economical alternative to feeder cell irradiation for T-cell expansion
Source: Front Immunol. 2024 Sep 11;15:1453740. doi: 10.3389/fimmu.2024.1453740 (PMC11427811; doi:10.3389/fimmu.2024.1453740)
Supplement: Supplementary file 1 [file Presentation1.pdf]

Supplementary Figures

A.

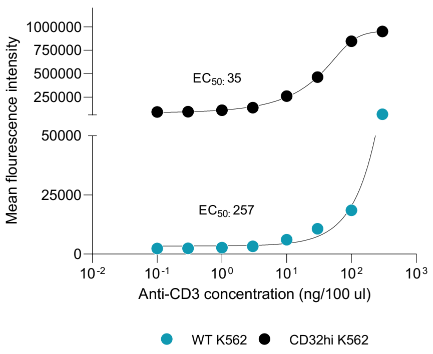

Figure S1. Binding affinity of CD32 K562 cells.
(A) Semi-logarithmic plot illustrating the binding affinity of CD32hi K562 cells relative to wild-type K562 cells.

A.

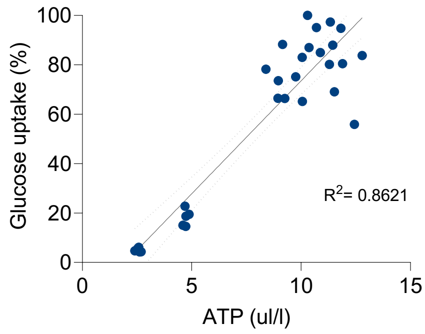

Figure S2. Association between glucose uptake and ATP.
(A) Scatter plot showing the correlation between glucose uptake and ATP levels.
Two independent experiments; n=30. R^2 between 0.7 and 0.9 considered highly correlated.

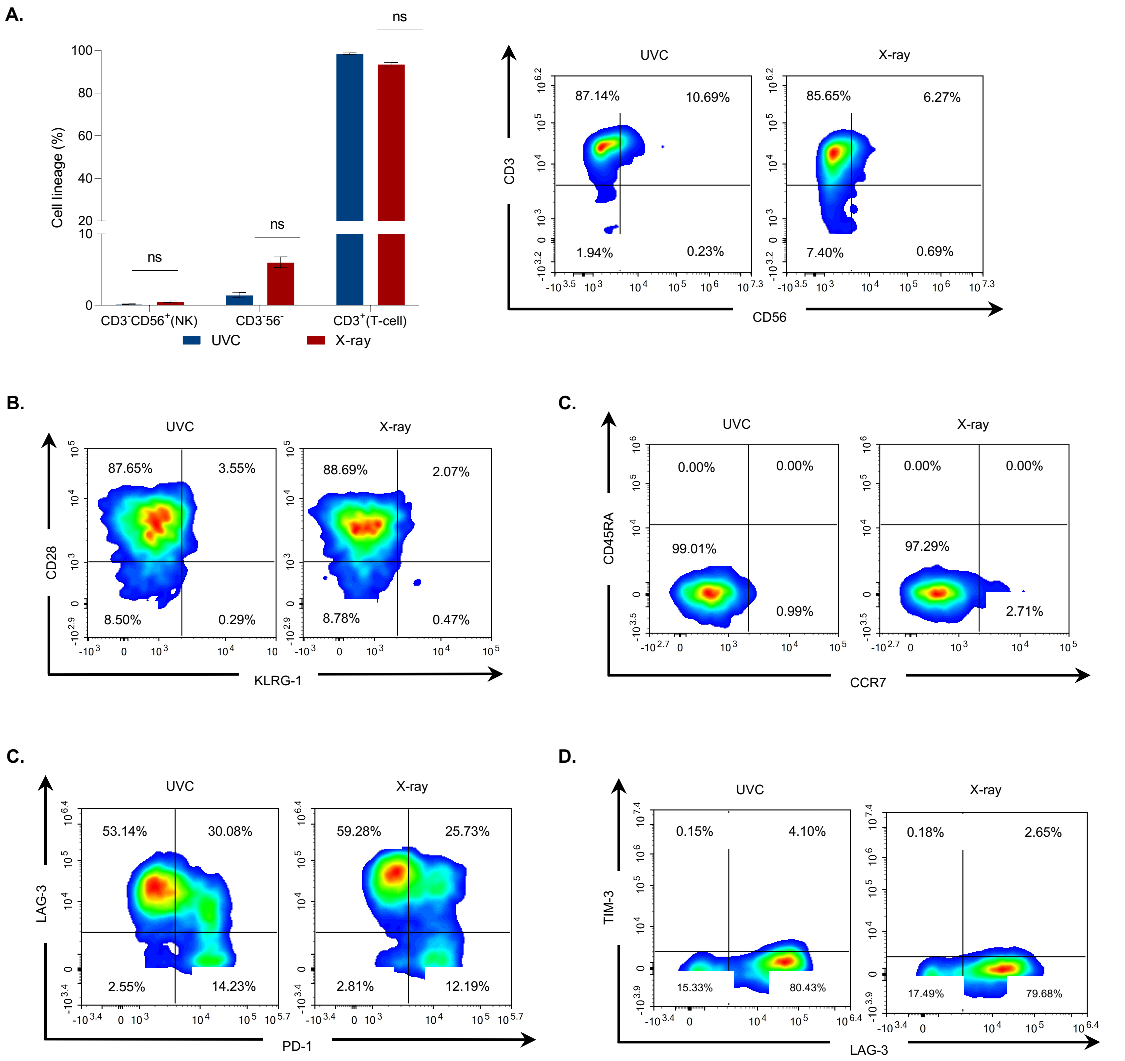

Figure S3. Representative flow cytometry plots of the immunophenotype of lung TILs (L002) expanded with UVC-irradiated PBMCs or X-ray-PBMCs.

(A) Bar graph showing the percentage of cell lineage in post-REP TILs (left) representative density plots depicting the percentage of each cell lineage.
(B) Representative density plots illustrating CD28 (Y-axis) and KLRG-1 (X-axis).
(C) Representative density plots showing T-cell memory phenotypes, CD45RA (Y-axis) and CCR7 (X-axis).
(D) Representative density plots displaying LAG-3 (Y-axis) and PD-1 (X-axis).
(E) Representative density plots presenting TIM-3 (Y-axis) and LAG-3 (X-axis).
Data are demonstrated as mean ± SEM. Two independent experiments; n=4, per group. Two-tailed Student's t-test, ns; not significant.

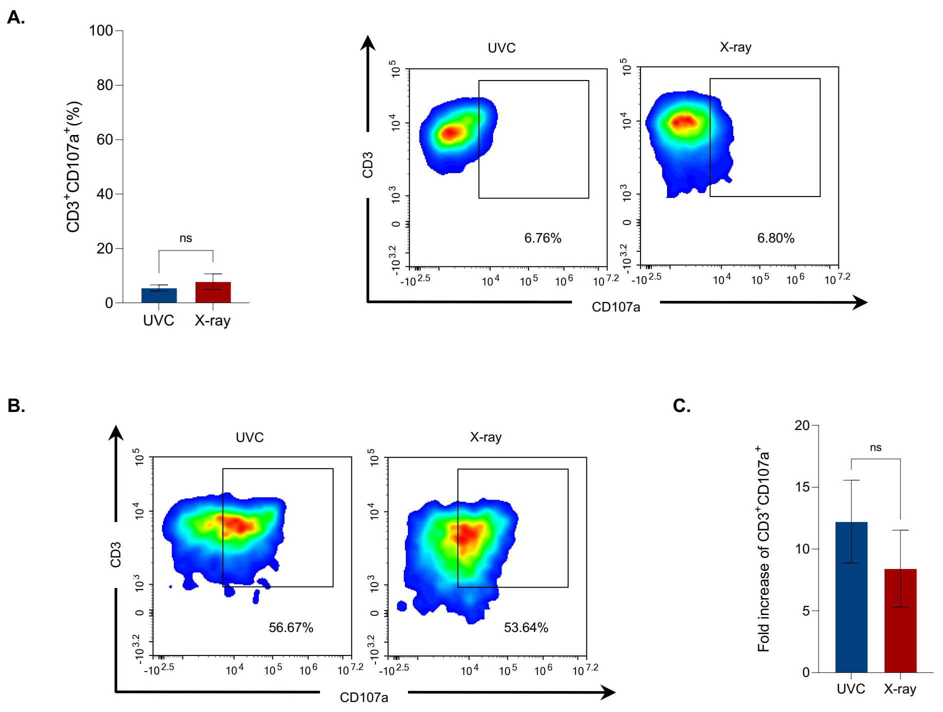

FigureS4. Effector function of TILs expanded with irradiated PBMCs.
(A) Bar graph displaying the percentage of $CD3^+CD107a^+$ TIL subset in un-stimulated condition (left) and representative density plot of $CD3^+CD107a^+$ population in unstimulated post-REP lung TILs (L002).
(B) Representative plots of $CD3^+CD107a^+$ post-REP lung TIL (L002) following re-stimulation with anti-human CD3 and CD28.
(C) Bar graph showing a fold increase in $CD3^+CD107a^+$ subset after stimulation with anti-human CD3 and CD28.
Data are demonstrated as mean ± SEM. Two independent experiments; n=4, per group. Two-tailed Student's t-test, ns; not significant.

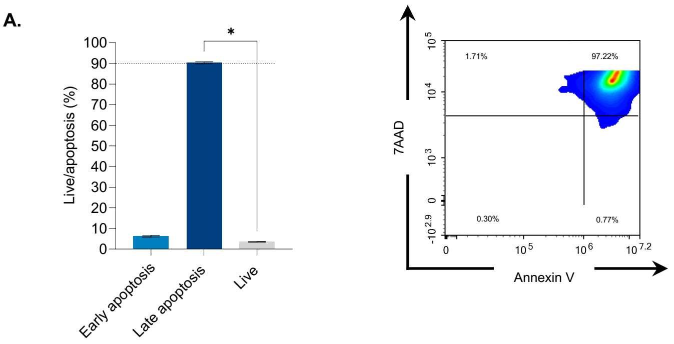

Figure S5. Characteristics of PBMCs following UVC irradiation.
(A) Bar graph demonstrating the percentage of live and apoptotic PBMCs on day 11 after UVC irradiation (left) and representative density plot displaying live and apoptosis cells, defined by 7AAD⁻Annexin-V⁻ and 7AAD⁺ or/and Annexin V⁺, respectively (right). Data are demonstrated as mean ± SEM. Two independent experiments; n=4, per group. Two-tailed Student's t-test; *P ≤ 0.05.

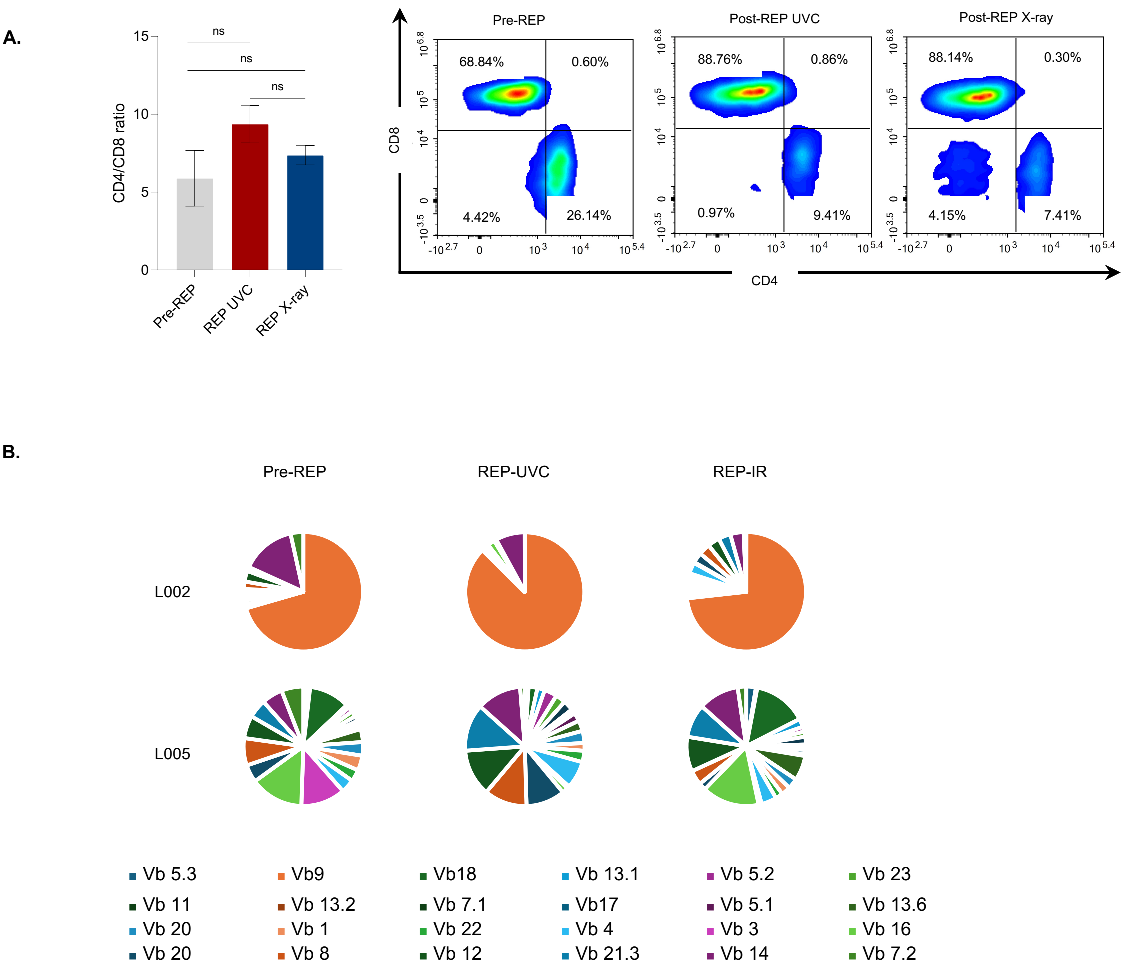

Figure S6. Distribution of CD4, CD8, and TCR Vβ families in TIL expanded with irradiated PBMCs.
(A) Bar graph illustrating the ratio of CD4 and CD8 before and after TIL expansion (left) and representative density plot displaying the percentage of CD4 and CD8 in pre- and post-REP L002 lung TILs (right).
(B) Pie charts displaying the frequency of individual Vβ families in two lung TILs (L002; top, L005; bottom) .
